# Novel Genetic Risk Variants Associated with Oral Tongue Squamous Cell Carcinoma

**DOI:** 10.1007/s12105-025-01784-0

**Published:** 2025-04-25

**Authors:** Rayan Nikkilä, Antti Mäkitie, Heikki Joensuu, Saara Markkanen, Klaus Elenius, Outi Monni, Aarno Palotie, Elmo Saarentaus, FinnGen, Tuula Salo, Argyro Bizaki-Vallaskangas

**Affiliations:** 1 Department of Otorhinolaryngology - Head and Neck Surgery, University of Helsinki and HUS Helsinki University Hospital, Helsinki, Finland; 2 Finnish Cancer Registry, Institute for Statistical and Epidemiological Cancer and Research, Helsinki, Finland; 3 Research Program in Systems Oncology, Faculty of Medicine, University of Helsinki, Helsinki, Finland; 4 Department of Oncology, HUS Helsinki University Hospital and University of Helsinki, Helsinki, Finland; 5 Department of Otolaryngology, Faculty of Medicine and Health Technology, Tampere University, Tampere, Finland; 6 The Wellbeing Services County of Pirkanmaa, Tampere, Finland; 7 Institute of Biomedicine, and MediCity Research Laboratory, University of Turku, Turku, Finland; 8 Turku Bioscience Centre, University of Turku and Åbo Akademi University, Turku, Finland; 9 Department of Oncology, Turku University Hospital, Turku, Finland; 10 iCAN Digital Precision Cancer Medicine Flagship, University of Helsinki, Helsinki, Finland; 11 Applied Tumor Genomics Research Program, Faculty of Medicine, University of Helsinki, Helsinki, Finland; 12 Institute for Molecular Medicine Finland and the Helsinki Institute of Life Science, University of Helsinki, Helsinki, Finland; 13 The Stanley Center for Psychiatric Research and Program in Medical and Population Genetics, The Broad Institute of MIT and Harvard, Cambridge, MA USA; 14 Analytic and Translational Genetics Unit, Department of Medicine, Department of Neurology, and Department of Psychiatry, Massachusetts General Hospital, Boston, MA USA; 15 Department of Oral and Maxillofacial Diseases, University of Helsinki, Helsinki, Finland; 16 Translational Immunology Research Program, Faculty of Medicine, University of Helsinki, Helsinki, Finland; 17 Department of Pathology, HUS Helsinki University Hospital, Helsinki, Finland; 18 Research Unit of Population Health, Faculty of Medicine, University of Oulu, Oulu, Finland; 19 Medical Research Center, Oulu University Hospital, Oulu, Finland

**Keywords:** Tongue cancer, Genetic variant, Single nucleotide polymorphism, FinnGen, Genome-wide association

## Abstract

**Purpose:**

Limited data from genome-wide association studies (GWAS) focusing on oral tongue squamous cell carcinoma (OTSCC) are available. The present study was conducted to explore genetic associations for OTSCC.

**Methods:**

A GWAS on 376 cases of OTSCC was conducted using the FinnGen Data Freeze-12 dataset. The case-cohort included 205 males and 171 females. Cases with malignancies involving the base of the tongue or lingual tonsil were excluded from the case-cohort. Individuals with no recorded history of malignancy were used as controls (n = 407,067). A Phenome-wide association study (PheWAS) was performed for the lead variants to assess their co-associations with other cancers.

**Results:**

GWAS analysis identified three genome-wide significant loci associated with OTSCC (*p* < 5 × 10–8), located at 5p15.33 (rs27067 near gene LINC01511), 10q24 (rs1007771191 near RPS3AP36), and 20p12.3 (rs1438070080 near PLCB1), respectively. PheWAS showed associations of rs27067 mainly with prostate cancer (OR = 1.06, *p* = 5.41 × 10^–7^), and seborrheic keratosis (OR = 1.11, *p* = 1.51 × 10^–11^). A co-directional effect with melanoma was also observed (OR = 0.93, *p* = 6.24 × 10^–5^).

**Conclusion:**

The GWAS detected two novel genetic associations with OTSCC. Further research is needed to identify the genes at these loci that contribute to the molecular pathogenesis of OTSCC.

**Supplementary Information:**

The online version contains supplementary material available at 10.1007/s12105-025-01784-0.

## Introduction

Oral squamous cell carcinoma (OSCC) accounts for approximately 80% of all malignant tumors in the oral cavity, with the tongue being the most affected site, particularly in developed nations [1, 2]. The incidence of oral tongue squamous cell carcinoma (OTSCC) among young adults (< 45 years of age) has risen globally, although the underlying causes for this trend remain uncertain [3]. In addition to tobacco and alcohol consumption, which remain the primary risk factors for oral cancer, betel quid chewing and processed meat are also recognized as risk factors [4]. Unlike oropharyngeal cancer, tongue cancer is infrequently associated with human papilloma virus (HPV) infection [2]. However, OTSCC can develop in individuals without any known risk factors, suggesting a significant role for genetic susceptibility and gene-environment interactions in oral carcinogenesis, particularly among young patients [3, 5]. For instance, polymorphisms in alcohol-related genes, such as *ADH1B* (alcohol dehydrogenase 1B) and *ADH7* (alcohol dehydrogenase 7), have already been linked to the disease [6]. Associated germline mutations are understudied. Instead, environmental factors, such as tobacco and alcohol consumption, may lead to somatic mutations or epigenetic changes that play a more prominent role in the pathogenesis [7].

Genome-wide association studies (GWAS) examine millions of genetic variants across multiple genomes to identify those that are statistically associated with specific phenotypes. This approach has yielded numerous strong associations for various traits and conditions, including cancer, with the number of linked variants expected to rise as GWAS sample sizes continue to expand. [8] However, relatively few GWAS studies have focused on oral cancer. A GWAS comprising 6,034 oral cavity and pharyngeal cancer cases and 6,585 controls from Europe, North America, and South America detected four loci associated (*p* < 5 × 10^–8^) specifically with oral cancer [9]. These included two novel regions at 2p23.3 (containing e.g. *GPN1*) and 9q34.12 (*LAMC3*), as well as two previously known cancer loci at 9p21.3 (*CDKN2B-AS1*) and 5p15.33 (*CLPTM1L*). A Taiwanese study [10] confirmed association of previously identified loci at 5p15.33 (*TERT-CLMPT1L*), 4q23 (*ADH1B),* 6p21.32 (HLA-DQ gene cluster), 6p21.33 (HLA-B), 9p21.3 (*CDKN2B-AS1*), and 9q34.12 (*LAMC3*) with oral cancer, and further identified two novel independent loci at 6p21.32 (*SKIV2* and *TNXB*). Specific methylation changes associated with OTSCC have also been reported [11].

Considering the distinct environmental exposures around the world, as well as the genetic diversity among different ethnicities, we hypothesize that there are both shared genetic susceptibility loci and loci that are unique to defined single populations for oral cancer. To date, large-scale GWASs on oral cancer remain limited, and none have specifically focused on OTSCC. Although the anatomical proximity of different subsites in the oral cavity may suggest they could be treated as a single entity, cancers in these subsites represent distinct diseases with varied etiological, biological, and histological characteristics. Indeed, gene expression differences associated with head and neck squamous cell carcinoma aggressiveness have been reported to be highly site-specific. [12] Even subsite-specific differences in gene expression have been reported for OSCC [13]. In this study, we conducted a GWAS using the FinnGen database to identify novel genetic risk variants associated with OTSCC, which is the most frequently encountered tumor subsite in the oral cavity.

## Materials and Methods

Genotype data of participants were obtained from FinnGen study release 12. FinnGen (accessible at finngen.fi/en) is a collaborative public–private research initiative that integrates genomic data from 480,000 individuals in Finland (as of release 12) with their digital healthcare records. FinnGen involves collaboration between Finnish biobanks, associated institutions (such as universities and university hospitals), global pharmaceutical industry partners, and the Finnish biobank cooperative, FINBB.

In the present study, we included data from the 2024 Release (Release 12), which comprised approximatively 480.000 post-QC samples, 520,210 (pre-QC samples), 520,210 individuals with endpoints, and 520,105 individuals with detailed longitudinal data. Information on disease diagnoses was obtained from the Care Register for Health Care (Finnish Institute of Health and Welfare) and the National Cause of Death Register provided by Statistics Finland.

### Study Population

**Fig. 1:**
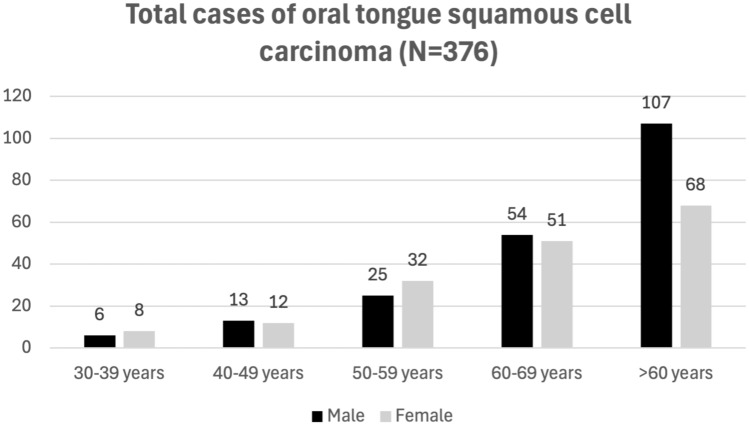
Demographics for cases wih OTSCC

Patients with OTSCC were identified using the International Classification of Diseases, the tenth and the ninth Revision (ICD-10 and ICD-9) codes C02.0, C02.1, C02.2, C02.3, C02.8, and C02.9. A total of 376 individuals with OTSCC were identified, 205 males and 171 females (Fig. 1). Cases with malignancies of the base of tongue (ICD-10: C01) or lingual tonsil (ICD-10: C02.4) were excluded from the case-cohort. Only patients with a diagnosis of OTSCC were included in the analysis. Individuals without record of malignancy were used as controls (n = 407,067).

A quantile–quantile plot of the observed versus expected χ^2^ test statistics did not show a large deviation from what was expected by chance (inflation factor λ = 0.99013). The great majority of the study population was of white ancestry.

### Genotyping and Quality Control

The detailed methods of the FinnGen study have been described by Kurki et al. [14]. Briefly, genotyping of the FinnGen participants’ peripheral venous blood samples was performed using Illumina and Affymetrix chip arrays (Illumina Inc., San Diego, CA, and Thermo Fisher Scientific, Santa Clara, CA). Samples were removed if they were duplicates, had ambiguous sex information, had high missing genotype data (> 5%), excessive heterozygosity (± 4 SD), or were of non-Finnish ancestry. After filtering, the FinnGen dataset release 12 included 473,681 individuals. Variants were excluded if they exhibited high missingness (> 2%), deviated significantly from Hardy–Weinberg equilibrium (*p* < 1 × 10^–6^), or had a minor allele count below three. Prephasing was performed using Eagle 2.3.5 with 20,000 conditioning haplotypes, and genotype imputation was done with Beagle 4.1, using the population-specific SISu v4.0 reference panel, which is based on GRCh38 coordinates and includes whole-genome sequences of 8,554 Finnish individuals. Variants were further excluded if the imputation INFO score was below 0.6 or the minor allele frequency was less than 0.0001.

### Genome-Wide Associations

The association analysis for imputed variants was conducted using Regenie version 2.2.4. To correct for population substructure, the outcome associations were tested using an additive model adjusted by sex, age, and the first ten principal components of the genetic data. In men, the non-PAR region of the X-chromosome was coded to reflect dosage compensation, where hemizygous men were treated equivalently to homozygous women. A genome-wide significance threshold was set at *p* < 5 × 10^–8^.

### Characterization of the Associated Loci

Associated loci were defined as genomic regions within a ± 1 Mb window around the primary variant. Each distinct locus included at least one genome-wide significant variant (*p* < 5 × 10⁻⁸) separated by a minimum of 1 Mb. Novel loci were identified according to the NHGRI-EBI catalog of human genome-wide association studies. Candidate genes within each new locus were prioritized based on their physical proximity to the index variant and existing literature regarding their biological function and clinical importance. Based on the NHGRI-EBI catalog of human genome-wide association studies, the locus was identified as novel. Genes within each new locus were prioritized for analysis based on their physical proximity to the index variant and existing literature regarding their biological function and clinical importance.

### Research Permission

Participants in the FinnGen study gave informed consent for biobank research in compliance with the Finnish Biobank Act. Separate research cohorts gathered before the Finnish Biobank Act (enacted in September 2013) and at the start of the FinnGen study (August 2017) were originally collected under study-specific consents. These cohorts were later transferred to Finnish biobanks following approval by the Finnish Medicines Agency (Fimea) and the National Supervisory Authority for Welfare and Health. Recruitment procedures adhered to the biobank protocols approved by Fimea.

The Coordinating Ethics Committee of the Hospital District of Helsinki and Uusimaa (HUS) issued the statement for the FinnGen study under Nr HUS/990/2017. Additionally, the FinnGen study received approvals from the Finnish Institute for Health and Welfare, under permit numbers THL/2031/6.02.00/2017, THL/1101/5.05.00/2017, THL/341/6.02.00/2018, THL/2222/6.02.00/2018, THL/283/6.02.00/2019, THL/1721/5.05.00/2019, and THL/1524/5.05/2019; Digital and population data service agencies (permit numbers: VRK43431/2017-3, VRK/6909/2018-3, VRK/4415/2019-3); the Social Insurance Institution (permit numbers: KELA 58/522/2017, KELA 131/522/2018, KELA 70/522/2019, KELA 98/522/2019, KELA 134/522/2019, KELA 138/522/2019, KELA 2/522/2020, KELA 16/522/2020); and Findata permit numbers THL/2364/14.02/2020. The Biobank Access Decisions for FinnGen samples and data used in FinnGen Data Freeze 11 include the following: THL Biobank BB2017_55, BB2017_111, BB2018_19, BB_2018_34, BB_2018_67, BB2018_71, BB2019_7, BB2019_8, BB2019_26, BB2020_1, BB2021_65; Finnish Red Cross Blood Service Biobank 7.12.2017; Helsinki Biobank HUS/359/2017, HUS/248/2020, HUS/150/2022 §12, §13, §14, §15, §16, §17, §18, and §23; Auria Biobank AB17-5154 and amendment #1 (August 17, 2020); and amendments BB_2021-0140, BB_2021-0156 (August 26, 2021; February 2, 2022), BB_2021-0169, BB_2021-0179, BB_2021-0161, AB20-5926 and amendment #1 (April 23, 2020) and its modifications (September 22, 2021); and Biobank Borealis of Northern Finland_2017_101.

The processing of sensitive data complies with Article 9(2)(j) of the GDPR and Article 6(1)(7) of the Data Protection Act V13.3/2023 (1050/2018), as Article 9(1) of the GDPR does not restrict data processing for scientific, historical research, or statistical purposes. The research was conducted in accordance with the principles of the Declaration of Helsinki.

## Results

**Fig. 2:**
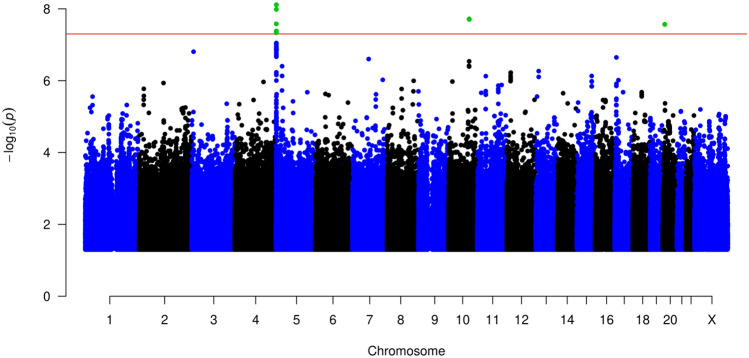
Manhattan plot for cases with OTSCC

We performed a GWAS analysis and identified three statistically significant loci associated with OTSCC with genome-wide significance *(p* < 5 × 10^−8^), located at 5p15.33, 10q24, and 20p12.3 (Fig. 2 and Table 1).

**Table 1:** Loci and lead variants associated with oral tongue squamous cell carcinoma

Chr	Position	Locus	rsid	Nearest gene	Ref. allele	Alt. allele	AF	*p*-value	OR (95% CI)
5	1,358,786	5p15.33	rs27067	*LINC01511*	C	T	47.65%	7.68 × 10^−9^	0.67 (0.58–0.76)
10	95,593,168	10q24.1	rs1007771191	*RPS3AP36*	C	G	0.09%	1.91 × 10^−8^	16.43 (6.19–43.63)
20	8,683,515	20p12.1	rs1438070080	*PLCB1*	A	C	0.02%	2.70 × 10^−8^	34.99 (9.99–122.55)

*AF* Allele frequency, *Alt* alternative, *Chr* chromosome, *OR* odds ratio, *CI* confidence interval, *Ref* reference, *rsid* Reference SNP cluster ID

**Fig. 3:**
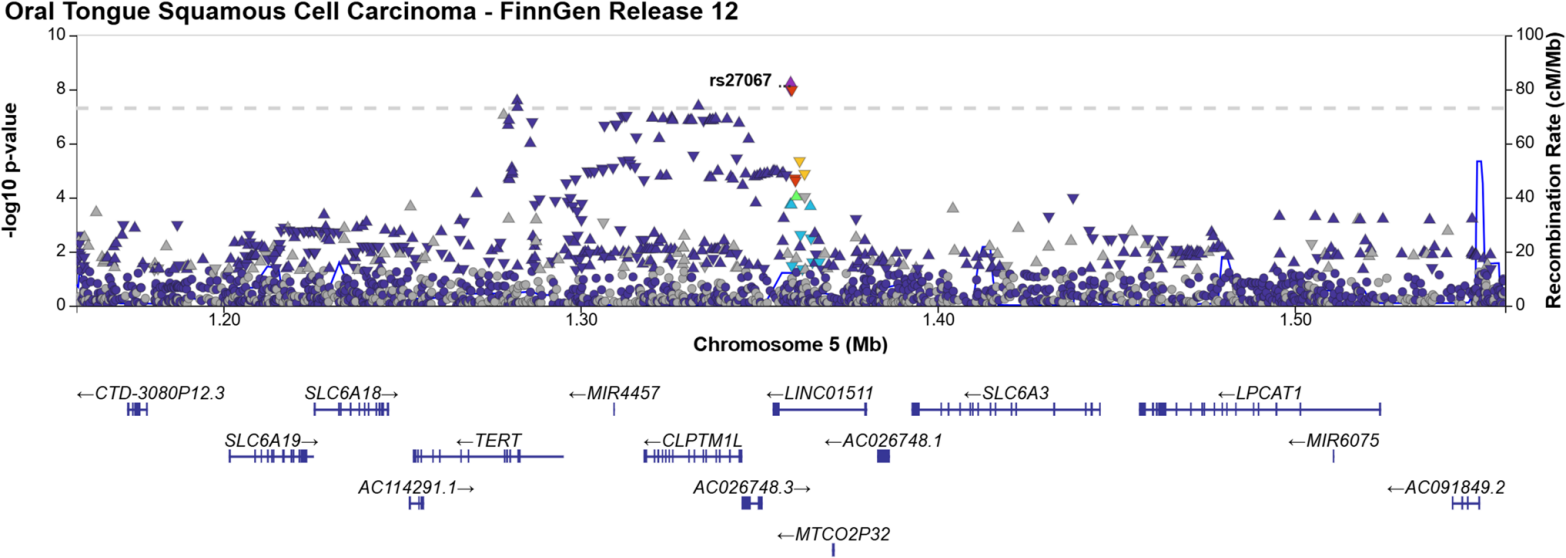
Regional association plot of OTSCC association on chromosome 5

**Fig. 4:**
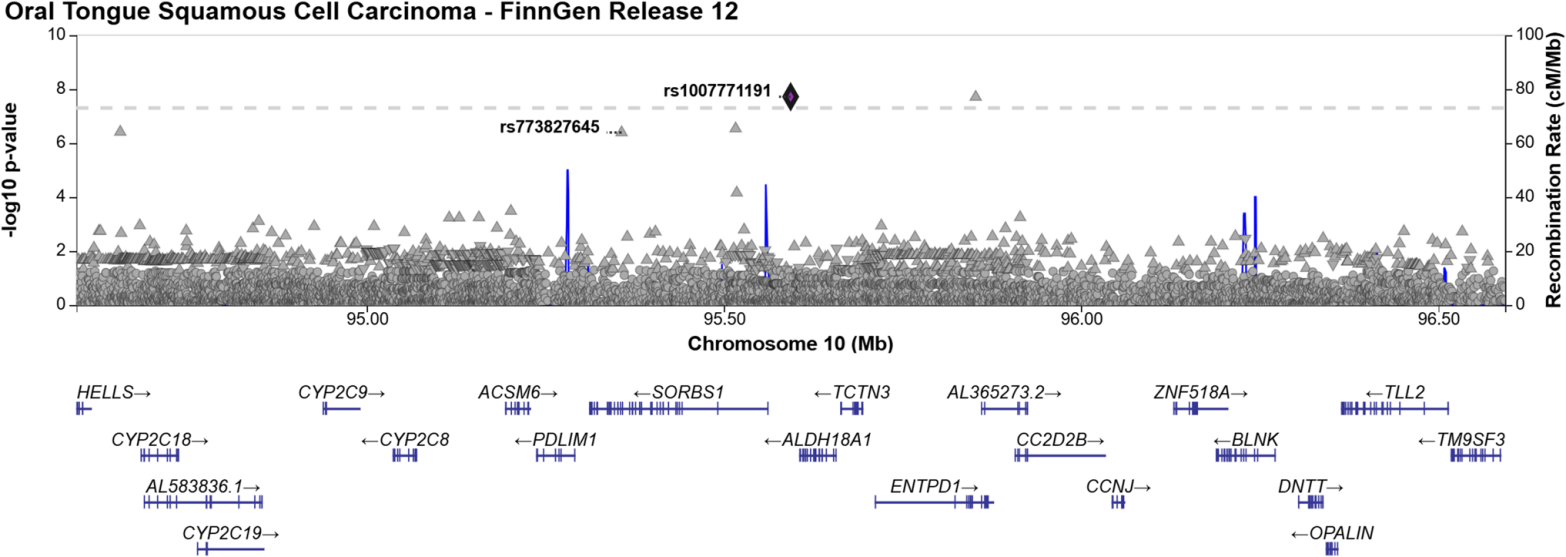
Regional association plot of OTSCC association on chromosome 10

**Fig. 5:**
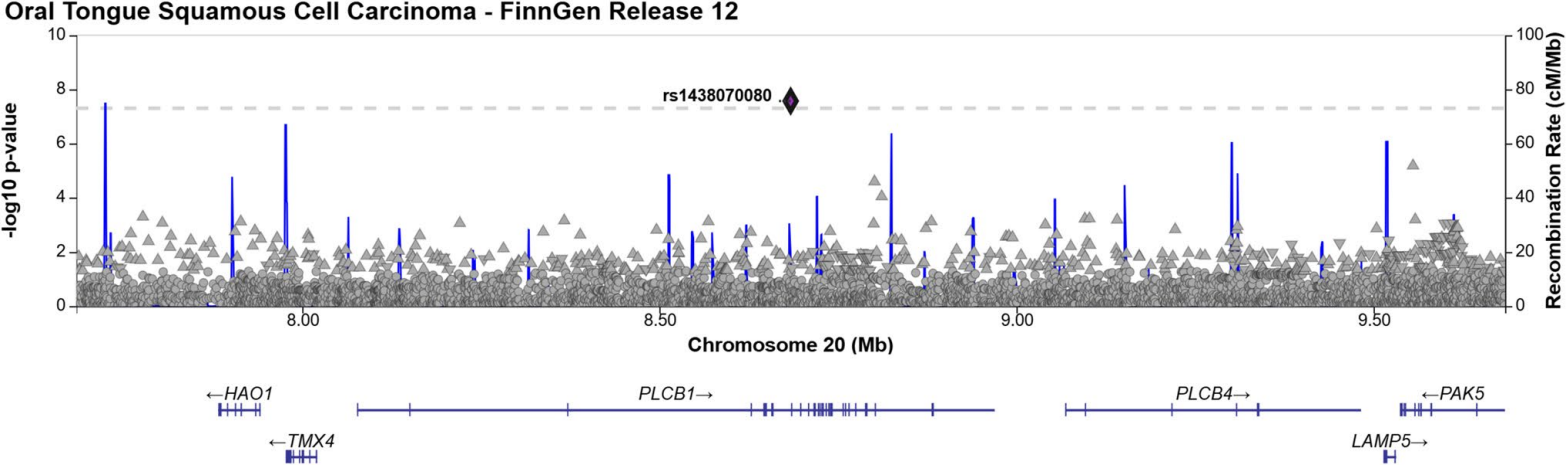
Regional association plot of OTSCC association on chromosome 20

The first locus identified mapped to 5p15.33, with the lead variant rs27067-T (AF = 47.65%, *p* = 7.68 × 10^−9^), an intergenic variant situated between *CLPTM1L* and *LINC01511* (long intergenic non-protein coding RNA 1511) (Fig. 3). Notably, several cancer-associated genes are located within a 1 Mb proximity including *CLPTM1L*, *TERT*, *BRD9*, *TRIP13*, *NKD2*, *LPCAT1*, and *IRX4* [14]. The second locus in chromosome 10 (10q24.1) harbored a novel variant (AF = 0.09%, *p* = 1.91 × 10^−9^) rs1007771191G near gene *RPS3AP36* (Fig. 4). The *SORBS1* missense variant rs773827645-C (AF = 0.089%, *p* = 4.04 × 10^−7^) was also in high linkage disequilibrium with rs1007771191 (r^2^ = 89.52%). Locus 10q24.1 also spans cancer-associated genes *ENTPD1* and *PDLIM1* [14]. The third locus was located in chromosome 20 (20p12.3) and included the *PLCB1* intron variant rs1438070080-C (AF = 0.02%, *p* = 2.70 × 10^−9^) (Fig. 5). The genome-wide significant variants at loci 10q24.1 and 20p12.3 are reported at lower allele frequencies among Non-Finnish Europeans at the Genome Aggregation Database (gnomAD) database (available at https://gnomad.broadinstitute.org), implicating Finnish enrichment.

### Phenome‑Wide Association Studies of the Lead Variants in the FinnGen Study

We assessed the co-directional effects of the identified lead variants with other cancers using data from the FinnGen study. Among the variants associated with OTSCC, rs27067 on 5p15.33 demonstrated significant associations with prostate cancer (OR = 1.06, *p* = 5.41 × 10^–7^), and seborrheic keratosis (OR = 1.11, *p* = 1.51 × 10^–11^). While prostate cancer and colorectal cancer showed an increased risk among T allele carriers, the effect of rs27067 was opposite in the case of OTSCC. PheWAS also revealed a co-directional effect with melanoma (OR = 0.93, *p* = 6.24 × 10^–5^). Although breast cancer did not show up in the PheWAS results of our study, the same locus on 5p15.33, and specifically the intron variant rs7726159 (AF = 32%, *p* = 2.62 × 10^–8^), has been reported in a previous GWAS study [15]. Variants rs1007771191 or rs1438070080 were not significantly associated with any cancer phenotype.

### Cross-Referencing with UKBB Results

To further validate our findings, we examined the associations of the identified variants with similar outcomes in publicly available UK Biobank (UKBB) data (available at https://pheweb.org/UKB-SAIGE/). The association of rs27067 with seborrheic keratosis was replicated in the UKBB (AF = 51%, *p* = 0.006). However, no significant association between the lead variant rs27067 at 5p15.33 and oral tongue cancer was reported in the UKBB database (*p* = 9.9 × 10⁻^1^). Variants rs1007771191 or rs1438070080 were not present in the UKBB database.

## Discussion

To our knowledge, we report the first comprehensive GWAS specifically for OTSCC. Our analysis confirmed the previously reported locus at 5p15.33, which is located near several genes implicated in cancer. Additionally, we identified two novel Finnish-enriched loci, at 10q24.1 and 20p12.1, which have not been previously associated with any phenotype. These loci also harbored several genes associated with cancer.

Despite the close anatomical relationship of various subsites within the oral cavity, they should not be treated as a single entity. Indeed, each subsite represents distinct disease features, differing in part in their etiology, biology, and histological features [12]. This constitutes the rationale for focusing specifically on OTSCC. GWAS offers a robust approach to uncovering the genetic background of diseases. Indeed, disease-associated loci identified by GWAS may inform about previously unrecognized biological pathways involved in disease mechanisms.

The association of the region 5p15.33 with OTSCC has been previously reported in European and Asian populations for oral cancer [9, 10]. Furthermore, in addition to oral cancer, several other cancers have been reported to be associated with the 5p15.33 region [16]. Potential associations, though non-significant, have been reported in the UK Biobank and FinnGen databases between the lead variant rs27067 and cancer phenotypes, including head and neck cancers. This variant is situated closest to *LINC01511*, a long-non-coding RNA. Long non-coding RNAs (lncRNAs) interact with DNA, RNA, or proteins to modulate various cellular processes, including cell growth, differentiation, and apoptosis. Thereby, lncRNAs are increasingly recognized as critical regulators in cancer development, by acting as tumor suppressors or oncogenes. [17] Several other cancer-associated genes are located within this locus. Among these, *CLPTM1L* (Cleft lip and palate transmembrane protein 1) and *TERT* (telomerase reverse transcriptase) may be the most interesting associations. *CLPTM1L* was originally identified in a screening search for genes that confer resistance to cisplatin [18]. *CLPTM1L* is often overexpressed in lung adenocarcinoma and its silencing increases cisplatin-induced apoptosis of tumor cells [19]. In the UK Biobank database, *CLPTM1L* was significantly associated with various cancer phenotypes, including lung, upper digestive tract, pancreatic, testicular, nasopharyngeal, and oral cancer. Overexpression of *CLPTM1L* has also been associated with poor prognosis of oral cancer patients [20–22] and cervical cancer recurrence [23].

*TERT* stands out due to its well-documented function in telomere maintenance and cancer development [24]. Under normal physiological conditions, *TERT* expression in adult humans is confined to the germ cells, transit-amplifying stem-like cells, and activated B and T cells. However, *TERT* promoter mutations and dysregulation have been implicated in several cancers, including those of the head and neck [25]. Indeed, telomerase reactivation occurs in approximately 85% of cancers [26]. Most head and neck squamous cell carcinomas show increased expression of *TERT* transcripts, which is associated with worse prognosis. In 2023, Boscolo-Rizzo et al. [27], published a meta-analysis in which they found that *TERT* promoter mutations were present in 21% of head and neck squamous cell carcinomas. The authors identified a significantly higher prevalence of these mutations in OSCC compared with other head and neck sites. Namely, in nearly half of OSCCs, *TERT* promoter mutations were found, while in oropharyngeal squamous cell carcinomas, their prevalence was as low as 1%, and that in larynx/hypopharynx as low as 12%. Moreover, the authors underlined those patients with head and neck squamous cell carcinoma carrying the − 124 C > T *TERT* promoter mutation in the tumors had more than double the risk of death and disease progression compared with patients whose tumors lacked this mutation.

The second significant locus, at 10q24.1, is marked by rs1007771191, a variant near *RPS3AP36*, a ribosomal pseudogene whose functional significance remains poorly understood. Notably, this locus also includes the missense variant rs773827645 in *SORBS1* (Sorbin and SH3 domain-containing protein 1). *SORBS1* encodes the CAP/Ponsin protein, involved among else in cell adhesion, cytoskeletal remodeling, and cell migration [28–31]. CAP/Ponsin is also involved in the regulation of glucose transport [32] and insulin signaling pathways [33]. Variants in the *SORBS1* gene have been associated with insulin resistance-related disorders in humans [34]. In breast cancer, the silencing of *SORBS1* promotes epithelial-to-mesenchymal transition and confers a loss of sensitivity to chemotherapeutic agents such as cisplatin by inhibiting the activity of p53 [35]. In prostate cancer, *SORBS1* was shown to be significantly downregulated, and might thus act as possible tumor suppressor role [36]. However, in vitro studies have also demonstrated that overexpression of *SORBS1* can enhance cancer cell migration, indicating its potential involvement in promoting cancer growth and metastasis [37]. *SORBS1* may thus contribute to cancer development by altering cell adhesion and migration processes, as well as by influencing metabolic pathways that support cancer development.

Other cancer-related genes located at the same locus include *ENTPD1* (Ectonucleoside triphosphate diphosphohydrolase-1), and *PDLIM1* (PDZ And LIM Domain 1). *ENTPD1*, also known as *CD39*, is an immune regulatory molecule in the tumor microenvironment through the breakdown of extracellular ATP and the production of adenosine. Cd39 is expressed on the surface of regulatory T cells (Tregs) and catalyzes the conversion of ATP and ADP into AMP. Subsequently, AMP is converted into adenosine, a strong immunosuppressor. Adenosine acts on its receptors on CD4 + , CD8 + T cells, and NK cells, thus inhibiting their functions and facilitating tumor growth [38]. Indeed, *CD39* is overexpressed in various human cancers, including head and neck cancers [39]. Experimental studies, both in vitro and in vivo with knockout mouse models, have demonstrated that inhibiting *CD39* effectively reactivates T-cell and NK-cell anti-tumor responses, facilitating the suppression of hepatic growth of metastatic melanoma tumors [40]. Currently, anti-Cd39 monoclonal antibodies are under investigation in various clinical trial settings, both as single agents and in combination regimens [38].

The *PDLIM1* gene encodes a protein involved in actin cytoskeleton organization [41] and in regulating signaling pathways, including the NF-κB pathway, which plays a critical role among others in inflammation, cancer cell proliferation, epithelial‑to‑mesenchymal transition, angiogenesis, and metastasis [42]. *PDLIM1*-deficient mice demonstrate increased levels NF-κB-mediated inflammation, which results in elevated production of pro-inflammatory cytokines and chemokines, which have been associated with cancer progression [43–45]. Expression of *PDLIM1* was significantly lower in colorectal cancer tissue samples compared with adjacent normal mucosal tissues. Furthermore, in vivo experiments using mouse models showed that loss of *PDLIM1* promotes invasiveness and metastasis in colorectal cancer, while overexpression inhibited the process. [46] Similar observations have been reported for hepatocellular cancer, where *PDLIM1* silencing promotes epithelial-to-mesenchymal transition and metastasis, whereas *PDLIM1* overexpression has the opposite effect [47]. However, in breast cancer mouse models, *PDLIM1* expression seems to increase during cancer progression [48].

The third locus, at 20p12.3, was marked by rs1438070080, an intronic variant near genes *PLCB1* (Phospholipase C Beta 1) and *PLCB4* (Phospholipase C Beta 4). These genes encode phospholipase C enzymes which are involved in intracellular transduction of many extracellular signals via regulation of calcium release from the endoplasmic reticulum. Plcb1 is mainly expressed in brain tissue, whereas Plcb4 is more ubiquitously expressed across various tissues, including the digestive tract. Mutations in the *PLCB1* gene have been associated with epileptic encephalopathy and West syndrome [49, 50]. Furthermore, *PLCB1* has been identified as an oncogenic driver, in cholangiocarcinoma [51], breast cancer [52], hepatocellular cancer [53], and gastric cancer [54]. Overexpression of Plcb1 has been correlated with advanced tumor stages and poorer survival outcomes in patients with breast, gastric cancers, and hepatocellular cancer, where it is thought to facilitate the migration and invasion of cancer cells. [52–54]. Similarly, dysregulation of *PLCB4* has been associated aggressive phenotypes in hepatocellular cancer and acute myeloid leukemia [55, 56]. Given the low allele frequency of rs1438070080 in OTSCC, it may represent a rare but high-impact variant. The association of this locus with two closely related signaling genes underscores its potential importance in OTSCC, warranting further functional studies to explore its exact role.

Although this study offers findings that could be valuable for future research, several limitations must be acknowledged. Firstly, GWASs tend to focus on common genetic variants, which may lead to missing rare but potentially impactful variants. Although adjustments were made for sex and age, other confounding factors, such as environmental influences, were not accounted for, which may impact the results. Furthermore, the findings could not be correlated with clinical data, such as cancer size, cancer HPV expression, and survival data. Lastly, restricting the study to participants of the Finnish ancestry limits genetic diversity, thus reducing the generalizability of the findings to other populations.

In summary, we identified three OTSCC susceptibility loci involving previously identified cancer-associated genes. Our findings highlight the importance of non-coding regions in cancer susceptibility. Variants in these regions could act as regulatory elements, influencing gene expression and downstream pathways involved in tumor initiation and growth. The loci identified in this study contain genes which have been implicated in telomere maintenance, immune evasion, and intracellular signaling, processes that are hallmarks of cancer [57]. Further validation of our findings in independent populations is essential. Furthermore, assessing whether the variations we uncovered are OTSCC-specific or shared between different head and neck cancer subsites would be valuable and constitutes the direction of our future research. Additionally, comparing variants between premalignant lesions and OTSCC would be highly relevant for understanding patient susceptibility to OTSCC.

## Supplementary Information

Below is the link to the electronic supplementary material.

Supplementary file1 (XLSX 40 KB)

## Data Availability

In accordance with national and European regulations, such as the General Data Protection Regulation (GDPR), access to sensitive individual-level health data requires approval from national authorities for specific research projects and approved researchers. The health data referenced here were obtained from national health registers, including the Finnish Institute for Health and Welfare, Statistics Finland, KELA, and the Digital and Population Data Services Agency, with approval granted either by these authorities or the Finnish Data Authority, Findata, for use in the FinnGen project. As study authors, we are unable to provide access to individual-level data. However, summary statistics from data releases will be made publicly available after a one-year embargo and can be accessed at finngen.fi/en/access_results.

## Code Availability

The association analysis for imputed variants was conducted using Regenie version 2.2.4.
